# Adiponectin/adiponectin receptor in disease and aging

**DOI:** 10.1038/npjamd.2015.13

**Published:** 2015-12-03

**Authors:** Masato Iwabu, Miki Okada-Iwabu, Toshimasa Yamauchi, Takashi Kadowaki

**Affiliations:** 1 Department of Diabetes and Metabolic Diseases, Graduate School of Medicine, The University of Tokyo, Tokyo, Japan; 2 PRESTO, Japan Science and Technology Agency, Kawaguchi, Japan; 3 CREST, Japan Science and Technology Agency, Kawaguchi, Japan

## Abstract

Adipocytes are not merely organs for energy conservation but endocrine organs secreting a wide array of physiologically active substances, i.e., adipokines. Of these adipokines, adiponectin is known to exert anti-diabetic and anti-atherosclerotic effects via adiponectin receptors (AdipoR)s, AdipoR1 and AdipoR2. Adiponectin has also recently been shown to regulate longevity signaling thus prolonging lifespan. Therefore, the strategy for activating adiponectin/AdipoR signaling pathways are expected to provide a solid basis for the prevention and treatment of obesity-related diseases such as the metabolic syndrome, type 2 diabetes and cardiovascular disease, as well as for ensuring healthy longevity in humans.

## Association between obesity and life expectancy

The evolutionary process of all forms of life on earth, including humans, can be seen as one of their adaptations over time to threats of starvation or malnutrition, and these environmental factors have remained determinants of their lifespan. In fact, infectious diseases associated with starvation, malnutrition and resultant decreased immunity has remained one major cause of death among humans worldwide. On the other hand, obesity has come to affect an increasing proportion of individuals worldwide, particularly in developed countries due to decreases in physical activity in their populations as a consequence of increases in industrial and societal automation in these countries.^1,2^ With insulin resistance as a background, obesity is shown to induce the so-called metabolic syndrome consisting of diabetes, dyslipidemia and hypertension, leading to increases in incidence of cardiovascular disease in affected individuals.^3,4^ Again, obesity has also been reported to be associated with cancer^5^ and Alzheimer’s disease,^6^ thus contributing to significant reductions in healthy life expectancy among modern humans.

## Role of adipokines in insulin resistance associated with obesity

In addition to their role in storing excess energy in the form of triglycerides, adipocytes have been shown, from the early 1990s onwards, to have roles as endocrine organs secreting numerous physiologically active substances called ‘adipokines’.^7–9^ The mechanisms through which obesity induces insulin resistance remained unclear for long. However, inflammatory adipokines such as TNF-α and MCP-1, during the course of adipocyte hypertrophy, were secreted in large amounts by adipocytes, and lead to impaired insulin signaling in the liver and skeletal muscle, thus inducing systemic insulin resistance.^10,11^ Although the presence of obesity is associated with decreases in the production/secretion of adiponectin, which has come to be characterized as a ‘beneficial’ adipokine.^12^

## Identification of adiponectin and its functions

Adiponectin has been identified independently by four groups through different approaches as a secreted protein expressed at high levels specifically in adipose tissue.^13–16^ Adiponectin has a molecular weight of about 30 kDa, consisting of four domains, i.e., an N-terminal signal sequence, a specific variable region containing cysteine residue essential for multimer formation, a collagen-like domain characterized by Gly–X–Y repeats, and a globular domain, and is classified, due to its structural features, as a member of the C1q family.^17^ Although crystal structure analysis of the globular domain demonstrates no homology to TNF-α in the primary amino-acid sequence, adiponectin is shown to be highly homologous to TNF-α in structure.^17^ The trimeric adiponectin as the minimal building block of adiponectin multimers is formed through hydrophobic interactions within the globular domain and is stabilized by the interactions of the collagen-like domains in a triple helix structure consisting of Gly-X-Y repeats. The assembly of adiponectin hexamers and multimers requires the formation of an intermolecular disulfide bond between cysteine residues conserved within the variable region.^18–20^

At the time that adiponectin was discovered, its functions remained largely unclear. In an effort to elucidate its functions, we compared, by using DNA chips, patterns of gene expression in the white adipose tissue of wild-type mice fed a high-fat diet versus mice with heterozygous peroxisome-proliferator-activated receptor-γ (PPAR-γ) deficiency in which adipocyte enlargement remains suppressed, and insulin sensitivity intact, even with high-fat diet, and found that adiponectin is abundantly expressed in small adipocytes, which suggested that adiponectin could represent a candidate adipocyte-derived insulin sensitizing factor. As the next step in the effort, we went on to examine whether administration of adiponectin might lead to improvements in insulin resistance in a mouse model of the metabolic syndrome. Although blood adiponectin levels are decreased, and insulin resistance and dyslipidemia are induced in KKAy mice on a high-fat diet, we demonstrated that insulin resistance and dyslipidemia are improved in these animals when treated with physiological doses of adiponectin.^21^ Of note, independently of our study, Lodish and his colleagues reported that a globular adiponectin, which is formed by proteolytic cleavage of full-length adiponectin, promotes fatty acid oxidation,^22^ and Scherer and his colleagues demonstrated that adiponectin increases insulin sensitivity and suppresses gluconeogenesis in the liver, thus lowering blood glucose levels.^23,24^ Thus, altogether, these experimental findings demonstrate that obesity leads to decreased adiponectin secretion thus causing insulin resistance and type 2 diabetes, whereas adiponectin represents an effective therapeutic option for insulin resistance and type 2 diabetes associated with obesity.

Following this discovery, analyses of adiponectin-deficient mice^25–28^ as well as adiponectin transgenic mice^29,30^ provided insights into the long-term effects of adiponectin, where adiponectin-deficient mice were shown to be associated with insulin resistance, impaired glucose tolerance, dyslipidemia, and hypertension, while adiponectin transgenic mice were shown to be associated with improvements in insulin resistance and diabetes mellitus.

## Pathophysiological implications for adiponectin in obesity-related diseases

On the one hand, adiponectin is shown to be available as varying multimers in the blood. Adiponectin multimers are primarily composed of low-molecular-weight trimers, middle-molecular-weight hexamers or even high-molecular-weight (HMW) multimers of a few hundred kDa.^18,19^ The HMW adiponectin has been shown to activate AMP kinase (AMPK) the most, of all forms of adiponectin.^31,32^ The scheme of the structure of adiponectin and various forms of multimers were provided previously.^33,34^ Again, adiponectin is shown to be regulated by a number of transcription factors, e.g., PPARγ,^35^ CCAAT-enhancer-binding protein (C/EBP) α^36^ and sterol regulatory element-binding protein 1c (SREBP1c).^37^ In addition, while thiazolidinediones (TZDs) are shown to directly increase the expression of adiponectin via PPARγ,^35^ Scherer and his colleagues further demonstrated that endoplasmic reticulum chaperones, e.g., ERp44 and Ero-1Lα, are involved as a mechanism through which TZDs promote HMW adiponectin secretion.^38^ Interestingly, it has also become clear that a fasted/starved state or caloric restriction leads to increases in blood levels of adiponectin. In this regard, Accili and his colleagues further clarified that activation of SIRT1^39,40^ leads to increases in blood levels of adiponectin as well as to improvements in hepatic insulin resistance in high-fat diet-fed mice,^41^ and that SIRT1 increases adiponectin or inhibits inflammatory cytokines by deacetylating PPARγ on Lys^268^ and Lys ^293^ (ref. 42).

On the other hand, the concentration of adiponectin is shown to be decreased in the presence of obesity and is inversely correlated with insulin resistance.^12^ It is also reported that the expression of adiponectin is increased in the adipose tissue of adipocyte-specific insulin receptor-deficient mice.^43^ Of related interest, blood levels of adiponectin are shown to be increased, despite the presence of severe insulin resistance, in individuals with inherited mutations in the insulin receptor gene.^44^ These findings suggest that insulin stimulation in adipose tissue may be responsible for decreased adiponectin expression. Again, it is shown that, in contrast to that in a starved state or caloric restriction, SIRT1 signaling is decreased, and blood adiponectin levels decreased, due to oxidative stress and inflammation, in obesity.^45^

There is an inverse correlation between circulating concentration of Adiponectin and not only obesity/insulin resistance but also type 2 diabetes mellitus. As for the evidence of association between adiponectin and abnormal glucose metabolism, prospective studies demonstrated that the greater the plasma concentration of adiponectin, the smaller the risk of onset of diabetes^46^ and that adiponectin may serve as the most powerful marker for the risk of onset of diabetes than glucose or insulin levels.^47^ In addition, it is also shown that the greater the HMW adiponectin concentration, the smaller the risk of onset of diabetes.^48^ As far as the evidence for association between adiponectin and abnormal lipid metabolism, the plasma level of adiponectin is shown to be positively correlated with the plasma level of HDL–cholesterol but inversely correlated with the plasma level of triglycerides in humans.^49^

Just as this systemic effect of adiponectin on glucose metabolism is thought likely to indirectly inhibit atherosclerosis, adiponectin is reported to directly inhibit atherosclerosis by acting on vascular endothelial cells and inflammatory cells. Indeed, adiponectin-deficient mice are shown to be associated with worsening of neointimal formation in response to vascular injury,^26,50^ while overexpression of globular adiponectin is shown to inhibit atherosclerosis in ApoE-deficient mice, a mouse model of atherosclerosis.^29^ In humans as well, high concentrations of adiponectin are shown to be associated with a significantly reduced risk for new onset of myocardial infarction, even after adjustment for other risk factors, and several reports are available in the literature to show that the greater the plasma concentration of adiponectin, the smaller the risk of onset of cardiovascular disease.^51–53^ Matsuzawa and his colleagues previously reported that male patients with hypoadiponectinemia (<4.0 μg/ml) had a twofold increase in coronary artery disease (CAD) prevalence, independent of well-known CAD risk factors and insisted that hypoadiponectinemia might be defined as a total adiponectin concentration <4.0 μg/ml.^52^ However, further studies seem to be necessary to obtain consensus. It is also known that ischemia/reperfusion-induced injury leads to increases in myocardial apoptosis and TNF-α expression, resulting in increases in size of myocardial infarction in adiponectin-deficient mice, and that adiponectin directly protects against myocardial injury.^54^

Furthermore, adiponectin is reported to be implicated not only in diabetes, dyslipidemia and hypertension as part of the metabolic syndrome, but in the onset of cancer associated with obesity. In this regard, clinical studies reported to date demonstrate that hypoadiponectinemia is associated with colorectal cancer,^55^ gastric cancer,^56^ endometrial cancer^57^ and breast cancer,^58^ and adiponectin is shown to inhibit the proliferation of various types of cancer,^59,60^ whereas the mechanisms involved remain yet to be elucidated and research findings on these mechanisms are eagerly awaited.

## Identification of AdipoRs and elucidation of their functions

We have succeeded in identifying adiponectin receptors (AdipoR1 and AdipoR2).^61^ Research shows that there is high homology between AdipoR1 and AdipoR2 (66.7% at the amino-acid level), which is known to be maintained across species from yeast to humans, where, interestingly, the yeast homolog (YOL002c) is shown to have a key role in fatty acid oxidation.^62^ AdipoR1 is expressed more or less ubiquitously but mainly in the skeletal muscle, whereas AdipoR2 is expressed particularly abundantly in the liver. One of the key facts about AdipoRs is that they are novel seven-transmembrane receptors whose topology is opposite to that of G-protein-coupled receptors (GPCRs), with an intercellular N terminus and an extracellular C terminus. We also confirmed through experiments using siRNA that AdipoR1 and AdipoR2 are both required for adiponectin binding to the cell membrane in cultured cells;^61^ subsequently we generated AdipoR1- and AdipoR2-knockout mice and found that adiponectin binding and function is abolished in AdipoR1/AdipoR2-double-knockout mice, thus demonstrating that AdipoRs represent key receptors in the body.^63^

We also found that AdipoR1/AdipoR2-double-knockout mice exhibit insulin resistance and impaired glucose tolerance, where the mechanisms involved include increased inflammation and oxidative stress as well as increased gluconeogenesis and decreased glucose uptake in organs essential for metabolism such as liver, skeletal muscle and adipose tissue.^63^ Again, we went on to show that the decreased expressions of AdipoR1 and AdipoR2 are partly responsible for diabetes, while restoring the adenovirus-mediated expression of AdipoR1 in the liver leads to activation of AMPK or restoring the expression of AdipoR2 leads to improvements in impaired glucose tolerance through activation of *PPARα*, acceleration of fatty acid oxidation, and through its anti-oxidant effects.^63^ It is reported that the expression of AdipoR1/R2 appears to be inversely regulated by insulin in physiological and pathophysiological states such as fasting/refeeding, insulin deficiency and hyperinsulinemia models via the insulin/phosphoinositide 3-kinase/Foxo1 pathway.^64^ Moreover, SNPs in adiponectin and AdiopRs are associated with various human diseases.^65^ Furthermore, microRNAs reportedly have critical roles in adiponectin regulation.^66^ However, further studies are necessary to make causative mechanisms clear. In addition, decreases in the expression of AdipoR1 and AdipoR2 were observed in people with a family history of type 2 diabetes mellitus and the expression levels of both receptors correlated positively with insulin sensitivity.^67^

## AdipoRs in signaling pathways for healthy longevity

Of the AdipoR1-mediated mechanisms through which adiponectin improves insulin resistance, we identified one which involves the activation in the liver and skeletal muscle of AMPK,^61,63,68,69^ which is a 5′AMP-activated serine–threonine kinase.^70^ In mammals, AMPK is known to regulate not only ATP-consuming (anabolic; e.g., lipogenesis, cholesterol synthesis, protein synthesis) but ATP-generating (catabolic; e.g., glycolysis, fatty acid oxidation) pathways and has thus come to be called a ‘metabolic sensor’ or a ‘fuel gauge’ involved in the regulation of intermediary metabolism in response to intracellular energy demand.^70^ AMPK initially drew attention in obesity and diabetes research, primarily because AMPK was thought to be implicated as a regulator promoting glucose utilization in skeletal muscle as induced by muscle contraction, as well as fatty acid oxidation. Reports followed, however, to demonstrate that the antidiabetic drug metformin activates AMPK^71^ as do leptin^72^ and adiponectin,^68^ and AMPK has thus taken center stage in obesity and diabetes research. Although AMPK is shown to be potently activated in exercise or fasting, which leads to increases in intracellular AMP concentrations, of the mechanisms through which AMPK becomes activated, to date, two distinct pathways are known: allosteric activation and AMPK kinase (AMPKK)-mediated activation. Of the AMPKKs involved in the latter, to date, Ca^2+^/calmodulin-dependent protein kinase kinase β (CaMKKβ) and LKB1 have been identified.^70,73,74^ In this regard, we demonstrated that the adiponectin/AdipoR1 pathway leads to AMPK activation, first, through the LKB1-dependent pathway as mediated by increases in AMP concentrations, and second, through the CaMKKβ-dependent pathway as mediated by increases in Ca^2+^ concentrations, thus clarifying that the adiponectin/AdipoR1 pathway represents an ‘exercise-mimicking’ signaling pathway.^69^ It remains to be determined whether exercise training could directly activate Adiponectin/AdpoR1 pathway signaling due to increase of expression of Adiponectin or AdipoR1, and it is of importance to be clarified.

In addition, we went on to demonstrate that, through SIRT1, activated AMPK activates PPARγ coactivator-1 α (PGC-1α),^69^ a molecule (cloned by Spiegelman and his colleagues who identified PPARγ as a major regulatory factor for adipocyte differentiation) initially reported to be expressed specifically in brown adipocytes^75^ but later reported by Spiegelman and his colleagues to be abundantly expressed in the skeletal muscle and liver as well. Known to be increasingly expressed in exercise, PGC-1α is deeply implicated in mitochondria synthesis, and PGC-1α transgenic mice are reported to be not only less susceptible to obesity or diabetes but also associated with prolonged life expectancy.^76^ It has now been clarified that once activated by adiponectin/AdipoR1 signaling, AMPK phospholyrates PGC-1α at Thr^177^ and Ser^538^ and regulates PGC-1α by deacetylating PGC-1α via SIRT1.^69^

In 2000, Sir2 was identified as a nicotinamide adenine dinucleotide (NAD)-dependent deacetylase required for regulation of lifespan in budding yeast,^77^ and the Sir2 ortholog was then shown to be essential for regulation of lifespan in nematodes and *Drosophila*, with more recent research suggesting a close relationship between regulation of aging/lifespan and regulation of metabolism.^39,78^ Indeed, SIRT1 has been reported to deacetylate PGC-1α NAD-dependently in the liver,^40^ an important organ responsible for gluconeogenesis critical for glucose homeostasis during fasting (starvation) as well as for setting the metabolic environment for individual organisms. SIRT1 expression is reported to be increased in the liver during fasting to upregulate the expression of gluconeogenic genes by facilitating SIRT1 binding to PGC-1α in a NAD-dependent fashion as well as by deacetylating lysine residue mainly at 13 points in PGC-1α.^40^ Later, SIRT1 has also been shown to deacetylate PGC-1α, thus regulating fatty acid oxidation in the skeletal muscle.^79^ As mentioned above, it is shown that once directly phospholyrated by AMPK, PGC-1α becomes activated and improves insulin resistance in the skeletal muscle. In this regard, of note, we clarified that adiponectin/AdipoR1 signaling activates PGC-1α by increasing the NAD:NADH ratio in the skeletal muscle thus activating SIRT1.^69^ Interestingly, deacetylation of PGC-1α is also noted in exercise, which is associated with AMPK and SIRT1 activation.^80^

It is also shown that, via AdipoR2, adiponectin upregulates the expression of the acyl-CoA-oxidase (ACO) gene involved in fatty acid oxidation as well as the uncoupling protein (UCP) genes involved in energy consumption.^61,63^ In an attempt to elucidate the mechanisms that lead to increases in the expression of ACO and UCPs, which have each a peroxisome proliferator response element (PPRE) in their promoter region, we found that intrinsic *PPARα* ligand activities are increased and the expression of the *PPARα* gene is also increased.^61^ Interestingly, it has also been clarified that the adiponectin/AdipoR2 pathway increases the expression of the catalase and superoxide dismutase (SOD) genes, thus alleviating oxidative stress in metabolic organs.^63^

It is well established that caloric restriction prolongs lifespan.^81^ The mechanisms involved include AMPK, mechanistic target of rapamycin (mTOR)^82^ and SIRT, which are also known as key longevity molecules. Lifespan is shown to be prolonged in the nematode *Caenorhabditis elegans* with overexpression of an AMPK-α subunit.^83,84^ Again, AMPK is known to inhibit protein synthesis by blocking mTOR signaling, thus inhibiting cancer cell growth or cancer-associated neoangiogenesis. Numerous reports published to date demonstrate that mTOR inhibition prolongs lifespan in yeast, nematodes and *Drosophila*, and the mTOR inhibitor rapamycin is also shown to prolong lifespan in mice.^82^

Quite apart from this, while tissue oxidative stress is increased in obesity, overexpression of the oxidative stress-detoxifying genes catalase and SOD is shown to be associated with prolonged lifespan.^85–87^

**Figure 1:**
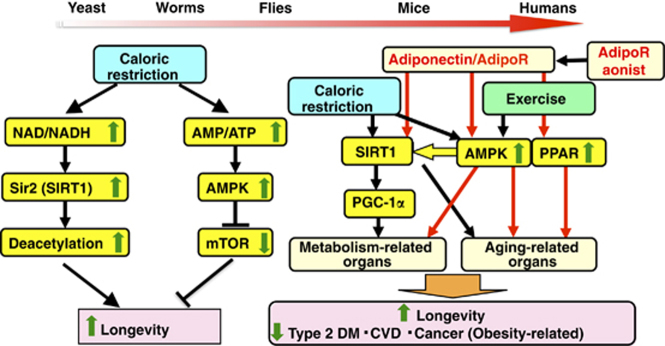
Caloric restriction, exercise and AdipoR pathway activate AMPK–SIRT1 and PPAR pathways, which may increase longevity in humans. Genetic studies using yeasts, worms and flies suggest that AMPK and SIRT1 pathways are implicated at the molecular level as signaling pathways that contribute to longevity through caloric restriction. SIRT1 deacetylates and activates transcription factor Foxo1 thus reducing stress responses, whereas AMPK prolongs lifespan by suppressing serine/threonine kinase mTOR, which regulates protein synthesis and cell cycle. In humans as well, adiponectin/AdipoR signaling is shown to activate the AMPK–SIRT1 pathway as well as to positively regulate the PPAR pathway thus alleviating oxidative stress in organs. Increased activation of adiponectin and AdipoRs pathways like exercise may have beneficial effects on healthy longevity and obesity-related diseases, such as type 2 diabetes, cardiovascular diseases (CVD) and cancers.

Given that adiponectin/AdipoR signaling is shown to activate the AMPK–SIRT1 pathway as well as to positively regulate the oxidative stress-detoxifying genes thus alleviating oxidative stress in tissues (Figure 1), we studied AdipoR-deficient mice on the assumption that their lifespan might be shortened. Quite interestingly, we found not only that lifespan was shortened in high-fat diet-fed AdipoR1-deficient and AdipoR2-deficient mice, but that it was most shortened in AdipoR1/AdipoR2-double knockout mice.^88^ The role of AdipoRs in longevity or metabolism in yeast, worm or flies largely remains to be clarified.

## Bringing about AdipoR agonists

Binging about changes in the metabolic capacity of individual organisms by enhancing adiponectin/AdipoR signaling also contributes to optimization of their metabolic environment. Thus, adiponectin/AdipoR-enhancing or adiponectin receptor-activating agents appear to have great potential as ‘exercise-mimicking’ agents capable of producing similar effects to those with exercise; indeed, their development has been eagerly awaited, not only as a novel avenue of definitive therapy for the metabolic syndrome, type 2 diabetes and atherosclerosis, but also as a viable therapeutic option for those incapable of exercise due to medical conditions or locomotor diseases. In this regard, we have succeeded in identifying orally active synthetic small-molecule AdipoR agonist (adiponectin receptor agonist; AdipoRon) by screening candidate compounds in the compound library at the University of Tokyo Drug Discovery Innovation Center.^88^ Subsequent research showed that AdipoRon improves metabolism in the liver, skeletal muscle and adipose tissue, and exerts anti-diabetic effects at the organism level, while it normalizes a shortened lifespan associated with obesity.^88^

## Future perspectives

In modern society, with obesity associated with overeating and lack of physical activity as a basis, the metabolic syndrome, diabetes, cardiovascular disease, cancer and Alzheimer’s disease are increasing to alarming proportions. Against this background, however, slowing down the process of aging, prolonging lifespan and maintaining youth appears to be feasible through optimal control of bodily responses to nutritional status, with research efforts focused on ensuring healthy longevity through control of metabolic regulatory pathways drawing attention worldwide. Indeed, since their identification, adiponectin and AdipoRs have emerged as potential targets in the management of lifestyle-related diseases, and GPCR research is being accelerated since the first structure of a human GPCR has been clarified in 2007 (ref. 89), given its immense implications for drug discovery.^90^ We have only recently succeeded in determining the crystal structures of human AdipoR1 and AdipoR2. The structures revealed their novel structural and functional properties, including the 7TM architecture, the zinc-binding site, which are completely distinct from those of GPCRs.^91^ This information will not only provide insights into signaling by the novel seven-transmembrane receptors AdipoRs but prove crucial to optimizing AdipoRon in its current form for use in humans (best-in-class). In the years to come, as candidate adiponectin receptor-activating small-molecule compounds are being fine-tuned as adiponectin receptor-activating agents for clinical use, it is hoped that their full potential will be realized as effective options for the prevention and treatment of lifestyle-related diseases for which obesity serves as a common basis.

## References

[bib1] Ng, M. et al. Global, regional, and national prevalence of overweight and obesity in children and adults during 1980-2013: a systematic analysis for the Global Burden of Disease Study 2013. Lancet 384, 766–781 (2014). 24880830 10.1016/S0140-6736(14)60460-8 PMC4624264

[bib2] Friedman, J. M. Obesity in the new millennium. Nature 404, 632–634 (2000). 10766249 10.1038/35007504

[bib3] Matsuzawa, Y. Pathophysiology and molecular mechanisms of visceral fat syndrome: the Japanese experience. Diabetes/metabolism reviews 13, 3–13 (1997). 9134345 10.1002/(sici)1099-0895(199703)13:1<3::aid-dmr178>3.0.co;2-n

[bib4] Reaven, G. Insulin resistance and coronary heart disease in nondiabetic individuals. Arteriosclerosis, thrombosis, and vascular biology 32, 1754–1759 (2012). 10.1161/ATVBAHA.111.241885 22815340

[bib5] Calle, E. E. , Rodriguez, C. , Walker-Thurmond, K. & Thun, M. J. Overweight, obesity, and mortality from cancer in a prospectively studied cohort of U.S. adults. N. Engl. J. Med. 348, 1625–1638 (2003). 12711737 10.1056/NEJMoa021423

[bib6] Kiliaan, A. J. , Arnoldussen, I. A. & Gustafson, D. R. Adipokines: a link between obesity and dementia? The Lancet. Neurology 13, 913–923 (2014). 25142458 10.1016/S1474-4422(14)70085-7 PMC4228955

[bib7] Kahn, C. R. Triglycerides and toggling the tummy. Nature Genet. 25, 6–7 (2000). 10802641 10.1038/75610

[bib8] Zhang, Y. et al. Positional cloning of the mouse obese gene and its human homologue. Nature 372, 425–432 (1994). 7984236 10.1038/372425a0

[bib9] Spiegelman, B. M. & Flier, J. S. Obesity and the regulation of energy balance. Cell 104, 531–543 (2001). 11239410 10.1016/s0092-8674(01)00240-9

[bib10] Hotamisligil, G. S. , Shargill, N. S. & Spiegelman, B. M. Adipose expression of tumor necrosis factor-alpha: direct role in obesity-linked insulin resistance. Science 259, 87–91 (1993). 7678183 10.1126/science.7678183

[bib11] Lazar, M. A. The humoral side of insulin resistance. Nat. Med. 12, 43–44 (2006). 16397562 10.1038/nm0106-43

[bib12] Arita, Y. et al. Paradoxical decrease of an adipose-specific protein, adiponectin, in obesity. Biochem. Biophys. Res. Commun. 257, 79–83 (1999). 10092513 10.1006/bbrc.1999.0255

[bib13] Scherer, P. E. , Williams, S. , Fogliano, M. , Baldini, G. & Lodish, H. F. A novel serum protein similar to C1q, produced exclusively in adipocytes. J. Biol. Chem. 270, 26746–26749 (1995). 7592907 10.1074/jbc.270.45.26746

[bib14] Hu, E. , Liang, P. & Spiegelman, B. M. AdipoQ is a novel adipose-specific gene dysregulated in obesity. J. Biol. Chem. 271, 10697–10703 (1996). 8631877 10.1074/jbc.271.18.10697

[bib15] Maeda, K. et al. cDNA cloning and expression of a novel adipose specific collagen-like factor, apM1 (AdiPose Most abundant Gene transcript 1). Biochem. Biophys. Res. Commun. 221, 286–296 (1996). 8619847 10.1006/bbrc.1996.0587

[bib16] Nakano, Y. , Tobe, T. , Choi-Miura, N. H. , Mazda, T. & Tomita, M. Isolation and characterization of GBP28, a novel gelatin-binding protein purified from human plasma. J. Biochem. (Tokyo) 120, 802–812 (1996). 10.1093/oxfordjournals.jbchem.a021483 8947845

[bib17] Shapiro, L. & Scherer, P. E. The crystal structure of a complement-1q family protein suggests an evolutionary link to tumor necrosis factor. Curr. Biol. 8, 335–338 (1998). 9512423 10.1016/s0960-9822(98)70133-2

[bib18] Pajvan, U. B. et al. Structure-function studies of the adipocyte-secreted hormone Acrp30/adiponectin. Implications fpr metabolic regulation and bioactivity. J. Biol. Chem. 278, 9073–9085 (2003). 12496257 10.1074/jbc.M207198200

[bib19] Waki, H. et al. Impaired multimerization of human adiponectin mutants associated with diabetes. Molecular structure and multimer formation of adiponectin. J. Biol. Chem. 278, 40352–40363 (2003). 12878598 10.1074/jbc.M300365200

[bib20] Tsao, T. S. et al. Role of disulfide bonds in Acrp30/adiponectin structure and signaling specificity. Different oligomers activate different signal transduction pathways. J. Biol. Chem. 278, 50810–50817 (2003). 14522956 10.1074/jbc.M309469200

[bib21] Yamauchi, T. et al. The fat-derived hormone adiponectin reverses insulin resistance associated with both lipoatrophy and obesity. Nat. Med. 7, 941–946 (2001). 11479627 10.1038/90984

[bib22] Fruebis, J. et al. Proteolytic cleavage product of 30-kDa adipocyte complement-related protein increases fatty acid oxidation in muscle and causes weight loss in mice. Proc. Natl Acad. Sci. USA 98, 2005–2010 (2001). 11172066 10.1073/pnas.041591798 PMC29372

[bib23] Berg, A. H. , Combs, T. P. , Du, X. , Brownlee, M. & Scherer, P. E. The adipocyte-secreted protein Acrp30 enhances hepatic insulin action. Nat. Med. 7, 947–953 (2001). 11479628 10.1038/90992

[bib24] Combs, T. P. , Berg, A. H. , Obici, S. , Scherer, P. E. & Rossetti, L. Endogenous glucose production is inhibited by the adipose-derived protein Acrp30. J. Clin. Invest. 108, 1875–1881 (2001). 11748271 10.1172/JCI14120 PMC209474

[bib25] Maeda, N. et al. Diet-induced insulin resistance in mice lacking adiponectin/ACRP30. Nat. Med. 8, 731–737 (2002). 12068289 10.1038/nm724

[bib26] Kubota, N. et al. Disruption of adiponectin causes insulin resistance and neointimal formation. J. Biol. Chem. 277, 25863–25866 (2002). 12032136 10.1074/jbc.C200251200

[bib27] Ma, K. et al. Increased beta -oxidation but no insulin resistance or glucose intolerance in mice lacking adiponectin. J. Biol. Chem. 277, 34658–34661 (2002). 12151381 10.1074/jbc.C200362200

[bib28] Nawrocki, A. R. et al. Mice lacking adiponectin show decreased hepatic insulin sensitivity and reduced responsiveness to peroxisome proliferator-activated receptor gamma agonists. J. Biol. Chem. 281, 2654–2660 (2006). 16326714 10.1074/jbc.M505311200

[bib29] Yamauchi, T. et al. Globular adiponectin protected ob/ob mice from diabetes and apoE deficient mice from atherosclerosis. J. Biol. Chem. 278, 2461–2468 (2003). 12431986 10.1074/jbc.M209033200

[bib30] Combs, T. P. et al. A transgenic mouse with a deletion in the collagenous domain of adiponectin displays elevated circulating adiponectin and improved insulin sensitivity. Endocrinology 145, 367–383 (2004). 14576179 10.1210/en.2003-1068

[bib31] Kobayashi, H. et al. Selective suppression of endothelial cell apoptosis by the high molecular weight form of adiponectin. Circ. Res. 94, e27–e31 (2004). 14752031 10.1161/01.RES.0000119921.86460.37 PMC4374479

[bib32] Hada, Y. et al. Selective purification and characterization of adiponectin multimer species from human plasma. Biochem. Biophys. Res. Commun. 356, 487–493 (2007). 17368570 10.1016/j.bbrc.2007.03.004

[bib33] Kadowaki, T. & Yamauchi, T. Adiponectin and adiponectin receptors. Endocr. Rev. 26, 439–451 (2005). 15897298 10.1210/er.2005-0005

[bib34] Radjainia, M. , Wang, Y. & Mitra, A. K. Structural polymorphism of oligomeric adiponectin visualized by electron microscopy. J. Mol. Biol. 381, 419–430 (2008). 18614177 10.1016/j.jmb.2008.06.015

[bib35] Maeda, N. et al. PPARgamma ligands increase expression and plasma concentrations of adiponectin, an adipose-derived protein. Diabetes 50, 2094–2099 (2001). 11522676 10.2337/diabetes.50.9.2094

[bib36] Saito, K. et al. Regulation of gelatin-binding protein 28 (GBP28) gene expression by C/EBP. Biol. Pharm. Bull. 22, 1158–1162 (1999). 10598019 10.1248/bpb.22.1158

[bib37] Seo, J. B. et al. Adipocyte determination- and differentiation-dependent factor 1/sterol regulatory element-binding protein 1c regulates mouse adiponectin expression. J. Biol. Chem. 279, 22108–22117 (2004). 15037635 10.1074/jbc.M400238200

[bib38] Wang, Z. V. et al. Secretion of the adipocyte-specific secretory protein adiponectin critically depends on thiol-mediated protein retention. Mol. Cell. Biol. 27, 3716–3731 (2007). 17353260 10.1128/MCB.00931-06 PMC1899995

[bib39] Guarente, L. Sirtuins as potential targets for metabolic syndrome. Nature 444, 868–874 (2006). 17167475 10.1038/nature05486

[bib40] Rodgers, J. T. et al. Nutrient control of glucose homeostasis through a complex of PGC-1alpha and SIRT1. Nature 434, 113–118 (2005). 15744310 10.1038/nature03354

[bib41] Banks, A. S. et al. SirT1 gain of function increases energy efficiency and prevents diabetes in mice. Cell Metab. 8, 333–341 (2008). 18840364 10.1016/j.cmet.2008.08.014 PMC3222897

[bib42] Qiang, L. et al. Brown remodeling of white adipose tissue by SirT1-dependent deacetylation of Ppargamma. Cell 150, 620–632 (2012). 22863012 10.1016/j.cell.2012.06.027 PMC3413172

[bib43] Blüher, M. et al. Adipose tissue selective insulin receptor knockout protects against obesity and obesity-related glucose intolerance. Dev. Cell 3, 25–38 (2002). 12110165 10.1016/s1534-5807(02)00199-5

[bib44] Semple, R. K. et al. Elevated plasma adiponectin in humans with genetically defective insulin receptors. J. Clin. Endocrinol. Metab. 91, 3219–3223 (2006). 16705075 10.1210/jc.2006-0166

[bib45] Furukawa, S. et al. Increased oxidative stress in obesity and its impact on metabolic syndrome. J. Clin. Invest. 114, 1752–1761 (2004). 15599400 10.1172/JCI21625 PMC535065

[bib46] Li, S. , Shin, H. J. , Ding, E. L. & van Dam, R. M. Adiponectin levels and risk of type 2 diabetes: a systematic review and meta-analysis. JAMA 302, 179–188 (2009). 19584347 10.1001/jama.2009.976

[bib47] Lindsay, R. S. et al. Adiponectin and development of type 2 diabetes in the Pima Indian population. Lancet 360, 57–58 (2002). 12114044 10.1016/S0140-6736(02)09335-2

[bib48] Nakashima, R. et al. Decreased total and high molecular weight adiponectin are independent risk factors for the development of type 2 diabetes in Japanese-Americans. J. Clin. Endocrinol. Metab. 91, 3873–3877 (2006). 16882743 10.1210/jc.2006-1158

[bib49] Zoccali, C. et al. Adiponectin, metabolic risk factors, and cardiovascular events among patients with end-stage renal disease. J. Am. Soc. Nephrol. 13, 134–141 (2002). 11752030 10.1681/ASN.V131134

[bib50] Matsuda, M. et al. Role of adiponectin in preventing vascular stenosis. The missing link of adipo-vascular axis. J. Biol. Chem. 277, 37487–37491 (2002). 12138120 10.1074/jbc.M206083200

[bib51] Ouchi, N. et al. Novel modulator for endothelial adhesion molecules: adipocyte-derived plasma protein adiponectin. Circulation 100, 2473–2476 (1999). 10604883 10.1161/01.cir.100.25.2473

[bib52] Kumada, M. et al. Association of hypoadiponectinemia with coronary artery disease in men. Arterioscler. Thromb. Vasc. Biol. 23, 85–89 (2003). 12524229 10.1161/01.atv.0000048856.22331.50

[bib53] Hara, K. et al. Reduced adiponectin level is associated with severity of coronary artery disease. Int. Heart J. 48, 149–153 (2007). 17409580 10.1536/ihj.48.149

[bib54] Shibata, R. et al. Adiponectin protects against myocardial ischemia-reperfusion injury through AMPK- and COX-2-dependent mechanisms. Nat. Med. 11, 1096–1103 (2005). 16155579 10.1038/nm1295 PMC2828682

[bib55] Wei, E. K. , Giovannucci, E. , Fuchs, C. S. , Willett, W. C. & Mantzoros, C. S. Low plasma adiponectin levels and risk of colorectal cancer in men: a prospective study. J. Natl Cancer Inst. 97, 1688–1694 (2005). 16288122 10.1093/jnci/dji376

[bib56] Ishikawa, M. et al. Plasma adiponectin and gastric cancer. Clin. Cancer Res. 11, 466–472 (2005). 15701829

[bib57] Petridou, E. et al. Plasma adiponectin concentrations in relation to endometrial cancer: a case-control study in Greece. J. Clin. Endocrinol. Metab. 88, 993–997 (2003). 12629074 10.1210/jc.2002-021209

[bib58] Miyoshi, Y. et al. Association of serum adiponectin levels with breast cancer risk. Clin. Cancer Res. 9, 5699–5704 (2003). 14654554

[bib59] Ishikawa, M. et al. Adiponectin inhibits the growth and peritoneal metastasis of gastric cancer through its specific membrane receptors AdipoR1 and AdipoR2. Cancer Sci. 98, 1120–1127 (2007). 17459059 10.1111/j.1349-7006.2007.00486.x PMC11160031

[bib60] Sugiyama, M. et al. Adiponectin inhibits colorectal cancer cell growth through the AMPK/mTOR pathway. Int. J. Oncol. 34, 339–344 (2009). 19148467

[bib61] Yamauchi, T. et al. Cloning of adiponectin receptors that mediate antidiabetic metabolic effects. Nature 423, 762–769 (2003). 12802337 10.1038/nature01705

[bib62] Karpichev, I. V. , Cornivelli, L. & Small, G. M. Multiple regulatory roles of a novel Saccharomyces cerevisiae protein, encoded by YOL002c, in lipid and phosphate metabolism. J. Biol. Chem. 277, 19609–19617 (2002). 11916977 10.1074/jbc.M202045200

[bib63] Yamauchi, T. et al. Targeted disruption of AdipoR1 and AdipoR2 causes abrogation of adiponectin binding and metabolic actions. Nat. Med. 13, 332–339 (2007). 17268472 10.1038/nm1557

[bib64] Tsuchida, A. et al. Insulin/Foxo1 pathway regulates expression levels of adiponectin receptors and adiponectin sensitivity. J. Biol. Chem. 279, 30817–30822 (2004). 15123605 10.1074/jbc.M402367200

[bib65] Kaarniranta, K. et al. Adiponectin receptor 1 gene (ADIPOR1) variant is associated with advanced age-related macular degeneration in Finnish population. Neurosci. Lett. 513, 233–237 (2012). 22387454 10.1016/j.neulet.2012.02.050

[bib66] Ishida, M. et al. MicroRNA-378 regulates adiponectin expression in adipose tissue: a new plausible mechanism. PLoS One 9, e111537 (2014). 25379946 10.1371/journal.pone.0111537 PMC4224402

[bib67] Civitarese, A. E. et al. Adiponectin receptors gene expression and insulin sensitivity in non-diabetic Mexican Americans with or without a family history of Type 2 diabetes. Diabetologia 47, 816–820 (2004). 15105989 10.1007/s00125-004-1359-x

[bib68] Yamauchi, T. et al. Adiponectin stimulates glucose utilization and fatty-acid oxidation by activating AMP-activated protein kinase. Nature med 8, 1288–1295 (2002). 12368907 10.1038/nm788

[bib69] Iwabu, M. et al. Adiponectin and AdipoR1 regulate PGC-1alpha and mitochondria by Ca(2+) and AMPK/SIRT1. Nature 464, 1313–1319 (2010). 20357764 10.1038/nature08991

[bib70] Kahn, B. B. , Alquier, T. , Carling, D. & Hardie, D. G. AMP-activated protein kinase: ancient energy gauge provides clues to modern understanding of metabolism. Cell Metab. 1, 15–25 (2005). 16054041 10.1016/j.cmet.2004.12.003

[bib71] Zhou, G. et al. Role of AMP-activated protein kinase in mechanism of metformin action. J Clin Invest 108, 1167–1174 (2001). 11602624 10.1172/JCI13505 PMC209533

[bib72] Minokoshi, Y. et al. Leptin stimulates fatty-acid oxidation by activating AMP-activated protein kinase. Nature 415, 339–343 (2002). 11797013 10.1038/415339a

[bib73] Hawley, S. A. et al. Calmodulin-dependent protein kinase kinase-beta is an alternative upstream kinase for AMP-activated protein kinase. Cell Metab. 2, 9–19 (2005). 16054095 10.1016/j.cmet.2005.05.009

[bib74] Woods, A. et al. Ca2+/calmodulin-dependent protein kinase kinase-beta acts upstream of AMP-activated protein kinase in mammalian cells. Cell Metab. 2, 21–33 (2005). 16054096 10.1016/j.cmet.2005.06.005

[bib75] Puigserver, P. et al. A cold-inducible coactivator of nuclear receptors linked to adaptive thermogenesis. Cell 92, 829–839 (1998). 9529258 10.1016/s0092-8674(00)81410-5

[bib76] Handschin, C. & Spiegelman, B. M. The role of exercise and PGC1alpha in inflammation and chronic disease. Nature 454, 463–469 (2008). 18650917 10.1038/nature07206 PMC2587487

[bib77] Imai, S. , Armstrong, C. M. , Kaeberlein, M. & Guarente, L. Transcriptional silencing and longevity protein Sir2 is an NAD-dependent histone deacetylase. Nature 403, 795–800 (2000). 10693811 10.1038/35001622

[bib78] Imai, S. & Guarente, L. NAD+ and sirtuins in aging and disease. Trends Cell Biol. 24, 464–471 (2014). 24786309 10.1016/j.tcb.2014.04.002 PMC4112140

[bib79] Gerhart-Hines, Z. et al. Metabolic control of muscle mitochondrial function and fatty acid oxidation through SIRT1/PGC-1alpha. EMBO J. 26, 1913–1923 (2007). 17347648 10.1038/sj.emboj.7601633 PMC1847661

[bib80] Cantó, C. et al. AMPK regulates energy expenditure by modulating NAD+ metabolism and SIRT1 activity. Nature 458, 1056–1060 (2009). 19262508 10.1038/nature07813 PMC3616311

[bib81] Colman, R. J. et al. Caloric restriction delays disease onset and mortality in rhesus monkeys. Science 325, 201–204 (2009). 19590001 10.1126/science.1173635 PMC2812811

[bib82] Laplante, M. & Sabatini, D. M. mTOR signaling in growth control and disease. Cell 149, 274–293 (2012). 22500797 10.1016/j.cell.2012.03.017 PMC3331679

[bib83] Apfeld, J. , O'Connor, G. , McDonagh, T. , DiStefano, P. S. & Curtis, R. The AMP-activated protein kinase AAK-2 links energy levels and insulin-like signals to lifespan in C. elegans. Genes Dev. 18, 3004–3009 (2004). 15574588 10.1101/gad.1255404 PMC535911

[bib84] Mair, W. et al. Lifespan extension induced by AMPK and calcineurin is mediated by CRTC-1 and CREB. Nature 470, 404–408 (2011). 21331044 10.1038/nature09706 PMC3098900

[bib85] Schriner, S. E. et al. Extension of murine life span by overexpression of catalase targeted to mitochondria. Science 308, 1909–1911 (2005). 15879174 10.1126/science.1106653

[bib86] Orr, W. C. & Sohal, R. S. Extension of life-span by overexpression of superoxide dismutase and catalase in Drosophila melanogaster. Science 263, 1128–1130 (1994). 8108730 10.1126/science.8108730

[bib87] Parkes, T. L. et al. Extension of Drosophila lifespan by overexpression of human SOD1 in motorneurons. Nature Genet. 19, 171–174 (1998). 9620775 10.1038/534

[bib88] Okada-Iwabu, M. et al. A small-molecule AdipoR agonist for type 2 diabetes and short life in obesity. Nature 503, 493–499 (2013). 24172895 10.1038/nature12656

[bib89] Rasmussen, S. G. et al. Crystal structure of the human beta2 adrenergic G-protein-coupled receptor. Nature 450, 383–387 (2007). 17952055 10.1038/nature06325

[bib90] Shimamura, T. et al. Structure of the human histamine H1 receptor complex with doxepin. Nature 475, 65–70 (2011). 21697825 10.1038/nature10236 PMC3131495

[bib91] Tanabe, H. et al. Crystal structures of the human adiponectin receptors. Nature 520, 312–316 (2015). 25855295 10.1038/nature14301 PMC4477036

